# Assessing the Architecture of *Drosophila mojavensis* Locomotor Evolution with Bulk Segregant Analysis

**DOI:** 10.1534/g3.119.400036

**Published:** 2019-03-29

**Authors:** Kyle M. Benowitz, Joshua M. Coleman, Luciano M. Matzkin

**Affiliations:** * Department of Entomology; ‡ Department of Ecology and Evolutionary Biology, and; § BIO5 Institute, University of Arizona, Tucson, AZ 85721, and; † Department of Biological Sciences, University of Alabama in Huntsville, Huntsville AL 35899

**Keywords:** advanced intercross lines, behavior, cactophilic, genetic mapping, larval activity

## Abstract

Behavior is frequently predicted to be especially important for evolution in novel environments. If these predictions are accurate, there might be particular patterns of genetic architecture associated with recently diverged behaviors. Specifically, it has been predicted that behaviors linked to population divergence should be underpinned by a few genes of relatively large effect, compared to architectures of intrapopulation behavioral variation, which is considered to be highly polygenic. More mapping studies of behavioral variation between recently diverged populations are needed to continue assessing the generality of these predictions. Here, we used a bulk segregant mapping approach to dissect the genetic architecture of a locomotor trait that has evolved between two populations of the cactophilic fly *Drosophila mojavensis*. We created an F8 mapping population of 1,500 individuals from advanced intercross lines and sequenced the 10% of individuals with the highest and lowest levels of locomotor activity. Using three alternative statistical approaches, we found strong evidence for two relatively large-effect QTL that is localized in a region homologous to a region of densely packed behavior loci in *Drosophila melanogaster*, suggesting that clustering of behavior genes may display relatively deep evolutionary conservation. Broadly, our data are most consistent with a polygenic architecture, though with several loci explaining a high proportion of variation in comparison to similar behavioral traits. We further note the presence of several antagonistic QTL linked to locomotion and discuss these results in light of theories regarding behavioral evolution and the effect size and direction of QTL for diverging traits in general.

Behavior has been traditionally thought to play an especially important role in adaptation to novel environments (Mayr 1963; West-Eberhard 1989). In particular, feeding, predator avoidance, mating, and reproductive behaviors have all been hypothesized to specifically promote survival when environments change (Wcislo 1989; Duckworth 2009). Frequently, this has been considered a consequence of the plasticity of these behaviors at an individual level (Ghalambor *et al.* 2007; Snell-Rood 2013), allowing organisms to adjust to unforeseen conditions (Wong and Candolin 2015). However, it has also been argued that the role of genetic behavioral variation in early adaptation is underappreciated (Duckworth 2009). Behavior is more labile than morphology or life-history on a phylogenetic scale (Blomberg *et al.* 2003) and has been demonstrated to evolve rapidly and subsequently mediate selection on traits such as morphology (McPeek 1995). If heritable behavior indeed plays a role in rapid adaptation to novel environments, this might be reflected in the genetic architecture of behavioral variation at both the intra- and interpopulation level.

In general, behaviors are considered to be highly polygenic and controlled mainly by loci of small effect (Flint and Mackay 2009). However, contrary to prior analysis a recent meta-analysis suggested that behavioral traits with predicted direct roles in speciation and environmental adaptation, notably courtship and feeding behavior, are underpinned by larger effect genes (York 2018). Furthermore, the average effect size of loci underlying behavioral variation increases as populations become more evolutionarily distant (York 2018). Beyond gene number and effect size, another potentially important characteristic of trait architecture between diverging populations are antagonistic QTL, where some alleles have opposite effects as those expected given the trait difference between parental populations (Rieseberg *et al.* 2002). Rieseberg *et al.* (2003) explicitly asked whether such effects might be more or less common in behaviors but could not locate enough data to address the question. However, antagonistic QTL have been found to be important components of complex behavior (Glater *et al.* 2014) and might be common for behaviors given their predicted high levels of trait correlations with other behaviors (Sih *et al.* 2004) and the fact that genes underlying behavior tend to be highly pleiotropic (Sokolowski 2001). To further address these questions, more empirical data on behaviors linked to recent evolutionary divergence is needed.

The cactophilic fly *Drosophila mojavensis* is endemic to the southwestern US and northwestern Mexico presents an ideal system for examining behavioral divergence on recent evolutionary timescales. Beginning 0.23-0.27 MYA, *D. mojavensis* has diverged from a population in Baja California (Smith *et al.* 2012) into four geographically, genetically and ecologically distinct populations (Heed 1978; 1982) in the Sonoran Desert, the Mojave Desert, Santa Catalina Island, and Baja California. Environmental variation across these four populations is broad and multifaceted. Each population primarily inhabits distinct species of host cacti (Sonora, *Stenocereus thurberi*; Baja California, *S. gummosus*; Santa Catalina Island, *Opuntia littoralis*; Mojave, *Ferocactus cylindraceus* [Heed 1978; 1982; Matzkin 2014]), of which many, especially those in different genera, display considerable microbial diversity and differentiation (Meyer *et al.* 1980; Kircher 1982; Starmer 1982; Starmer and Phaff 1983; Fogleman and Abril 1990) establishing the chemical and nutritional environments for *D. mojavensis* (Starmer *et al.* 1986). Further host-related environmental variables involve spatial and temporal properties of the necroses in which flies feed, which differ in size, abundance, duration, and distribution within and between plants (Mangan 1982; Etges 1989; Breitmeyer and Markow 1998). Beyond host plant, further climatic variables, notably temperature and humidity, differ between populations. Thus, the radiation of *D. mojavensis* into four host populations might potentially be linked to strong environmental selection on a wide array of behavioral traits, and behavioral evolution has been demonstrated for host preference (Newby and Etges 1998; Date *et al.* 2013; 2017; Crowley-Gall *et al.* 2016), courtship behavior (Etges *et al.* 2006) and larval behavior (Coleman *et al.* 2018). Here, we examine locomotor behavior, which displays large, heritable differences across populations (Coleman *et al.* 2018). Although it is not clear what the functional consequences or adaptive value of locomotor variation may be in *D. mojavensis* larvae, we have hypothesized that this may be a foraging related trait, as thought for similar phenotypic variation in *D. melanogaster* larvae (Sokolowski 1980). If so, larval locomotion could be related to host adaptation given the differences in distribution of nutrients across host cacti.

To assess the genetic architecture of population variation in *D. mojavensis* locomotor behavior, we performed a bulk segregant analysis. Bulk segregant analysis (BSA) maps quantitative traits by comparing allele frequencies between pools of individuals taken from the tails of the phenotypic distribution (Ehrenreich *et al.* 2010). This type of analysis has gained popularity in recent years due to its low cost and high power to detect both large and small effect variants. Bulk segregant analysis might be especially useful for hard to quantify traits such as behaviors, given that phenotypic values are needed only to assign individuals into pools and is therefore tolerant of a certain degree of measurement error. Sometimes referred to as extreme QTL analysis, BSA has been repeatedly used to corroborate other methodologies for unraveling genetic architecture, including for behaviors in *D. melanogaster* (Huang *et al.* 2012; Morozova *et al.* 2015; Shorter *et al.* 2015). However, BSA can be limited in its ability to detect linked QTL (Bastide *et al.* 2016), and often produces extremely wide peaks of significance (*e.g.*, Shorter *et al.* 2015). To attempt to mitigate these problems, we utilized three statistical methods to assess associations. We find robust evidence for two linked regions responsible for a large degree of the variation between our bulks, and moderate evidence for several other QTL throughout the genome that may further modulate locomotor behavior in *D. mojavensis*.

## Materials and Methods

### Flies used, phenotyping, and sample collection

We utilized single isofemale lines of genome sequenced *D. mojavensis* from Santa Catalina Island (Drosophila 12 Genomes Consortium 2007) and Guaymas, Sonora, Mexico (Allan and Matzkin 2019) in the laboratory. These populations were selected as having the lowest (Catalina Island) and highest (Sonora) larval speed among the four *D. mojavensis* populations (Coleman *et al.* 2018), allowing us to maximize the variance in the trait of interest. Additionally, limiting the number of parental genotypes used for a crossing design increases the ability to detect the effects of rare variants (Morozova *et al.* 2015). We maintained lines in the laboratory on banana-molasses media (Coleman *et al.* 2018) at 25°, 50% humidity, and a 14:10 light:dark cycle. The Catalina Island and Sonoran lines used here have been maintained in lab since 2002 and 1999, respectively; however, locomotor phenotypes measured in more recent collections are similar (Coleman *et al.* 2018), and we therefore do not expect inbreeding or laboratory adaptation to be responsible for phenotypic differences between these lines.

To generate the five replicate advanced intercross lines (AIL; Darvasi and Soller 1995), we placed 100 adult virgin males from Catalina Island and 100 adult virgin females from Sonora in glass bottles with banana-molasses media and allowed them to copulate and oviposit. We also performed the reciprocal cross in the same fashion. Upon reaching adulthood, we placed the F1 flies resulting from these crosses in cages (30cm × 30cm × 30cm; BugDorm, MegaView Science, Taichung, Taiwan) for ten days providing them with a fresh petri dish of banana-molasses media every two days. We then placed a petri dish of banana-molasses media covered with yeast (to induce oviposition) in the cage. After 24 hr, we transferred eggs from the plate into vials filled with 2-3 cm of the same media, placing roughly 40 eggs into each vial. We left these eggs to develop undisturbed and returned newly eclosed flies to new cages. This process was repeated for six additional generations, until we had obtained F8 flies. Additionally, this process was simultaneously replicated five times, creating five independent intercross lines.

To perform activity trials, we selected 1,500 F8 larvae at random during the third instar stage of development, 300 from each of the five intercross lines. In groups of five, we removed larvae from vials and placed them in a 10 cm petri dish filled with 1% agar, which was placed on top of an LED light pad (Huion, Shenzhen, China). We recorded trials using a Point Gray video camera (FLIR Systems, Wilsonville, OR, USA) for five minutes, retaining images every 2.5 sec. To avoid using a period of inactivity observed immediately after transferring larvae to the petri dish, we analyzed activity only during the last 50 sec before each larva reached the edge of the dish. We calculated the mean speed of each larva in cm/min during this 50 sec period using the TrackMate plugin for ImageJ (https://imagej.nih.gov/ij/). We obtained activity phenotypes for 1,484 larvae, with 16 discarded due to a complete lack of movement or other measurement discrepancies. Immediately after activity trials, we froze each larva whole at -80°. All trials were performed between 12:00 and 3:00 pm under identical conditions as described above. Following a previous BSA study of aggression in *D. melanogaster* (Shorter *et al.* 2015), we selected 150 individuals forming approximately the top and bottom 10% of the activity distribution for inclusion in the bulk segregant analysis. Each bulk contained individuals from all five replicate AIL populations. We used type I ANOVA in R to compare bulk means to the means of each parental population (Coleman *et al.* 2018).

### DNA extraction, sequencing, and bioinformatic analysis

We extracted DNA separately from each individual using Qiagen DNeasy spin columns (Qiagen, Venlo, Netherlands). We then quantified the DNA concentration of each sample using a NanoDrop (Thermo Fisher Scientific, Waltham, MA USA) and combined equal amounts of DNA from each sample into single pooled samples for low and high activity. Each bulk contained 4 μg of total DNA, and libraries for each were prepared using Illumina Truseq kits. Each sample was sequenced as paired end 150 bp reads using an Illumina NextSeq Mid Output Flowcell at the University of Arizona Genetic Core (UAGC) facility.

We trimmed raw reads for quality and adapter contamination using Trimmomatic (Bolger *et al.* 2014). We then mapped all remaining read pairs to the published *D. mojavensis* genome (Drosophila 12 Genomes Consortium 2007), which stems from the Catalina Island population, using bwa-mem (Li and Durbin 2009). We identified SNPs counted variants in each bulk using samtools mpileup (Li 2011) and VarScan (Koboldt *et al.* 2009). In order to remove any allele frequency bias created by using a reference genome from one of our source populations, we also mapped all reads to an aligned genome from the Sonoran population (Allan and Matzkin 2019), before counting SNPs as above. We summed the reference allele frequencies for each SNP across both datasets mapped to each reference, and only kept SNPs displaying a sum between 0.9 and 1.1, indicating equivalent results between reference genomes and therefore no mapping bias. For the remainder of the analysis, we used only the SNP counts from the data mapped to the Catalina Island reference genome. Before analysis, we performed further SNP filtering, removing SNPs with fewer than 100 total reads. We also limited our analysis to the ten largest scaffolds of the *D. mojavensis* genome, which contain 96.45% of the available genomic sequence and maps to all Muller elements (Schaeffer *et al.* 2008).

### Statistical analysis

We first analyzed allele frequency differences between the two bulks using the R package QTLseqr (Mansfeld and Grumet 2018), which performs a sliding window association analysis based on the G’ statistic proposed by Magwene *et al.* (2011). We chose a 500 kb window size to estimate G’. Significance of associated regions was controlled using FDR (Benjamini and Hochberg 1995) set to 0.01 following Magwene *et al.* (2011).

Next, we analyzed allele frequency differences on an individual SNP basis, using a Z-statistic as used in previous studies of *D. melanogaster* (Huang *et al.* 2012), calculated as $Z=(\rho_{1}-\;\rho_{2})/\sqrt{\rho_{0}(1-\rho_{0})(\frac{1}{n}+\frac{1}{c_{1}}+\frac{1}{c_{2}})}$. ρ_1_ and ρ_2_ are defined as the reference allele frequency in each bulk, ρ_0_ as the average reference allele frequency across bulks, n as the sample size of each bulk, and c_1_ and c_2_ as the depth of coverage for each bulk. We evaluated the significance of Z for each SNP using a standard normal distribution (Chevalier *et al.* 2014; Huang *et al.* 2012; Shorter *et al.* 2015), setting a significance threshold of *P* < 2.684x10^−8^ following Bonferroni correction of an initial threshold of *P* < 10^−5^ (Shorter *et al.* 2015). We performed calculations of Z and its significance in R 3.4.0.

Lastly, we analyzed our data using SIBSAM (Pool 2016), a set of perl scripts (https://github.com/JohnEPool/SIBSAM1) performing forward simulations to assess the likelihood that peaks in close physical proximity are actually separate QTL or artifacts due to linkage, and which could lead to overestimating QTL effect size. For this analysis, we used the same filtered set of individual SNPs as above. We defined non-overlapping windows to contain 50 SNPS each, generating an average window size of 8.1 kb to roughly match initial testing of SIBSAM (Pool 2016). We then took the average allele frequency difference for all SNPS in each window as our input to SIBSAM. Recombinational distance for chromosome 1 was taken from previous work to be 130.8 cM (Staten *et al.* 2004). To estimate recombination on the other chromosomes, we took chromosomal averages from *D. melanogaster* (Comeron *et al.* 2012) and adjusted them based on the recombination difference between the two species on chromosome 1; we thus used recombination rates that were 1.89 times higher than those in *D. melanogaster*, a rate on the conservative end of possible estimates for *D. mojavensis* (Ortiz-Barrientos *et al.* 2006). We assumed recombination to be uniform across each chromosome. We then ran SIBSAM using parameters for a single cross followed by eight generations of interbreeding, using 1,000 null simulations to assess the significance of primary peaks and shoulders. SIBSAM is designed to simulate results for the five major *D. melanogaster* chromosome arms, and therefore we excluded the dot chromosome (chromosome 6; Muller element F) from this analysis. Additionally, we stitched together separate chromosome scaffolds for chromosomes 1 and 4 to facilitate chromosome-wide simulations of recombination.

### Data availability

Raw reads are available at NCBI Bioproject PRJNA498146 (https://ncbi.nlm.nih.gov/sra/PRJNA498146). Behavioral data are publicly available at OSF (https://doi.org/10.17605/OSF.IO/JXVZW). Supplemental figures are available at figshare. Supplemental material available at Figshare: https://doi.org/10.25387/g3.7840256.

## Results

**Figure 1:**
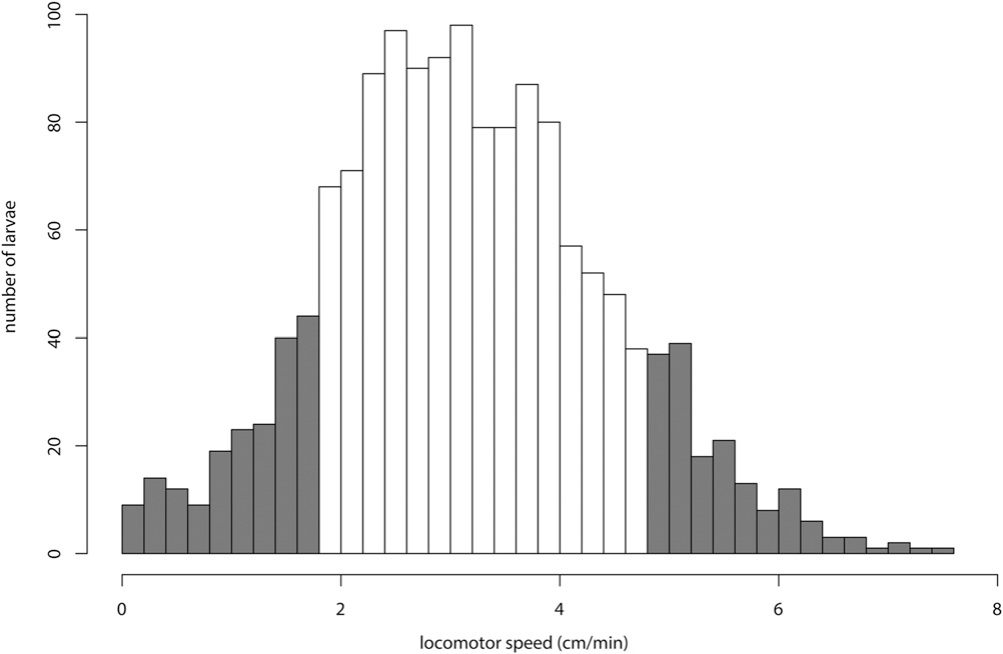
Distribution of larval locomotor phenotypes. Shaded regions represent roughly the areas of the distribution sampled for pooled sequencing.

The complete distribution of F8 *D. mojavensis* larval locomotor phenotypes is presented in Figure 1. Average locomotor speed across the entire dataset was 3.16 ± 1.28 cm/min (mean ± SD). Average locomotor speed for larvae sequenced in the “slow” bulk was 1.03 ± 0.45 cm/min, whereas that of those sequenced in the “fast” bulk was 5.46 ± 0.56 cm/min. Locomotor speed was significantly different between the bulks (F_1,298_ = 5745.0; *P* < 0.001). These means are also significantly more extreme than the trait means for both the parental Catalina Island line (2.48 ± 1.12 cm/min; F_1,201_ = 172.4; *P* < 0.001) and the Sonoran desert line (3.84 ± 1.39 cm/min; F_1,250_ = 166.2; *P* < 0.001), indicating that the advanced intercross lines have captured the natural range of phenotypic expression for this trait.

**Figure 2:**
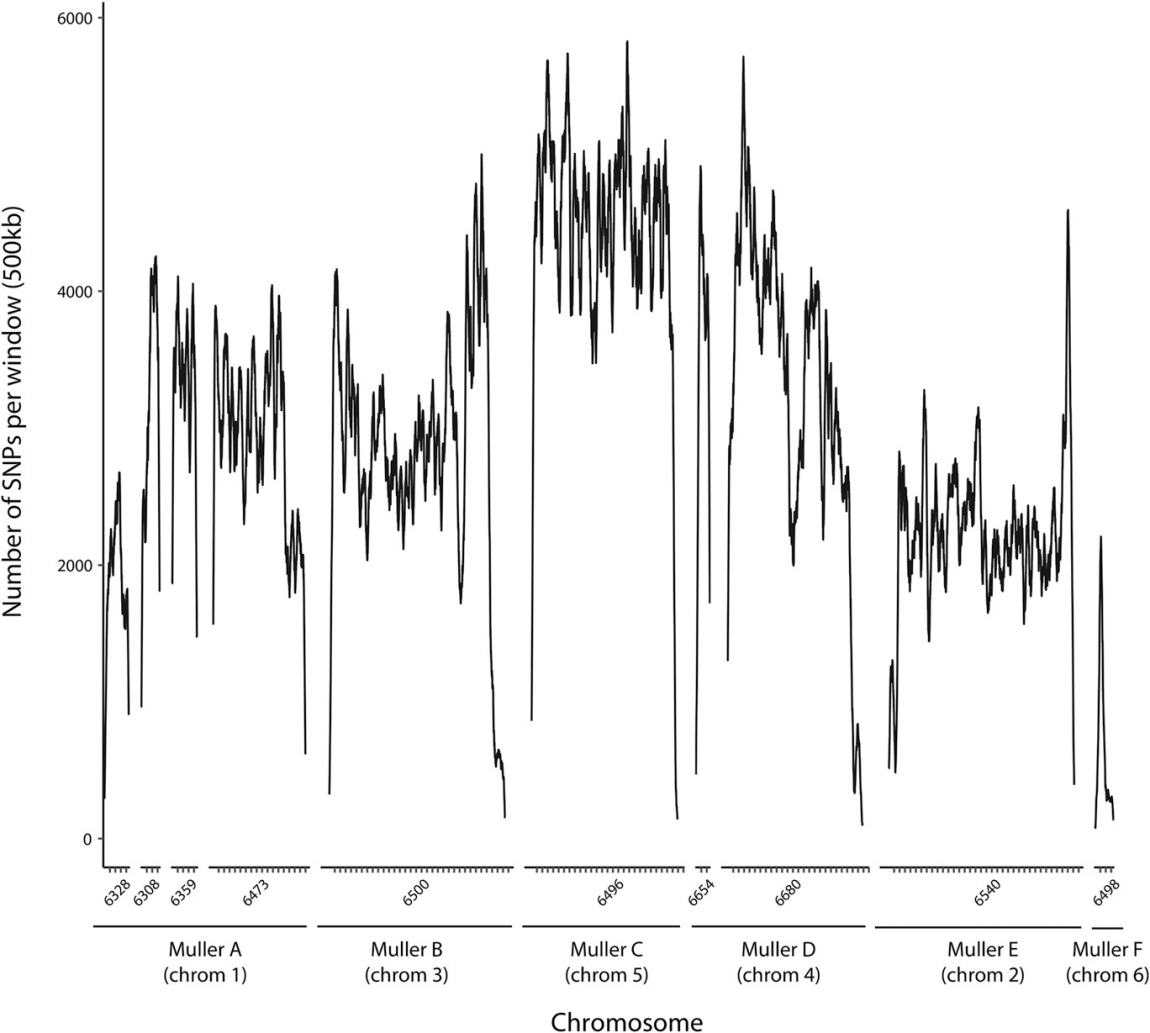
SNP density across the genome, presented in 500kb windows as used in G’ analysis. Ticks on the x axis represent 1 mb. Numbers below the x axis correspond to Flybase scaffold identifiers.

We obtained an initial set of 1,598,192 SNPs segregating in the five replicate AIL populations and analyzed 931,281 SNPs after filtering. The distribution of SNPs across the genome is presented in Figure 2. Average read depth across analyzed SNPs was 208.35 for the fast bulk, and 197.89 for the low bulk.

**Figure 3:**
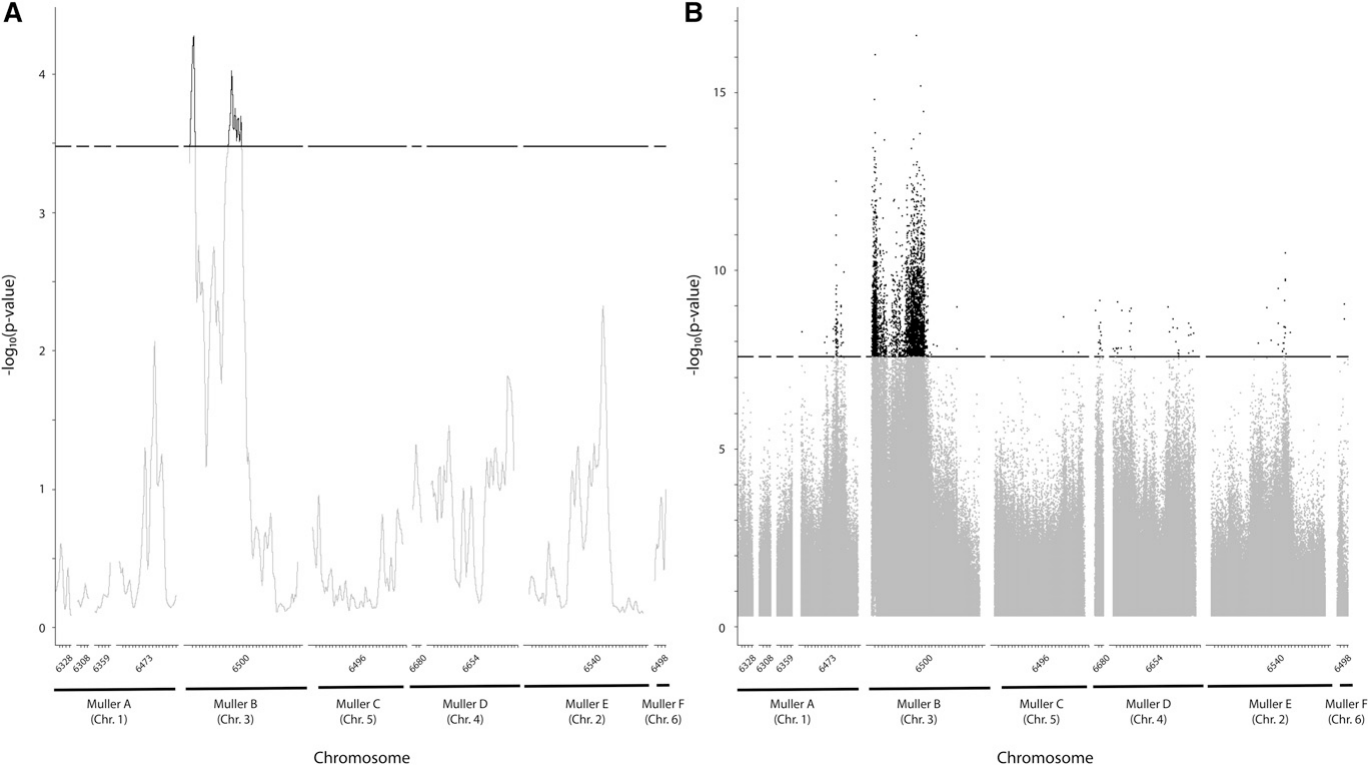
Association plots for each of the 10 *D. mojavensis* scaffolds analyzed. Ticks on the x axis represent 1 mb. A: Sliding window G’ analysis. The black bar indicates the threshold for QTL significance after FDR correction to 0.01. B: Individual SNP analysis with a Z-statistic. The black bar indicates the threshold for significance after Bonferroni correction to 10^−5^.Numbers below the x axis correspond to Flybase scaffold identifiers.

Sliding window analysis using a G’ statistic identified a large region on the first half of scaffold 6500 (chromosome 3) as significantly associated with phenotypic variation between our bulks (Figure 3A). Outside of this region, no other region on any other chromosome remotely approached FDR significance by this methodology. However, it is not clear whether this region should be considered to include one or two QTL; while it does appear that a valley separates two distinct regions (bp 169,299-1,714,039; bp 11,628,191-15,682,389), the allele frequency differences in this valley are still nearly significant themselves.

To complement the analysis presented above, we analyzed the same dataset on an individual, SNP-by-SNP basis. The same region identified in the sliding window analysis on chromosome 3 remained the most notable region of association (Figure 3B). Of 3,893 total SNPs passing a conservative Bonferroni correction, 3,745 were located to this chromosome, including the SNPs with by far the strongest statistical support (Figure 3B). All six chromosomes showed SNPs with evidence of associations.

**Figure 4:**
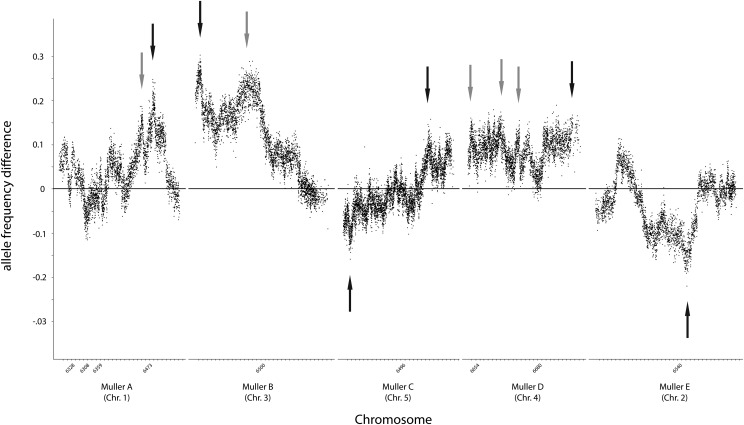
Allele frequency difference between the high and low pools for each 50 SNP window analyzed by SIBSAM. Positive values indicate a preponderance of the Sonoran desert allele in the high locomotion bulk, indicating the expected direction of the association. Black arrows mark primary peaks identified as significant by SIBSAM, whereas gray arrows indicate secondary peaks identified by SIBSAM to be distinct from nearby primary peaks based on additional simulations. Numbers below the x axis correspond to Flybase scaffold identifiers.

Lastly, we assessed our data using a markedly different approach, performing null simulations of statistical associations given our experimental design and an estimated recombination rate. This methodology suggested eleven significant QTL (Table 1; Figure 4), located across all five analyzed chromosomes. Simulations suggested that the broad region of association indicated by other analyses on chromosome 3, is in fact likely to consist of multiple independent QTL.

**Table 1:** Significant QTL identified by SIBSAM, with estimates of width and effect sizes

Chromosome scaffold #	QTL peak (bp)	QTL range (bp)	Median effect size	Effect size range (5^th^ – 95^th^ percentile)
scaffold 6473	7,863183	7,856377 – 7,951,477	0.098	0.034 – 0.152
scaffold 6473	10,475,325	9,145,800 – 10,791,875	0.113	0.086 – 0.192
scaffold 6540	22,259,909	20,438,927 – 23,000,330	0.128*	0.103 – 0.178
scaffold 6500	1,334,817	779,458 – 2,552,355	0.153	0.113 – 0.239
scaffold 6500	12,808,598	5,180,066 – 13,267,562	0.140	0.065 – 0.161
scaffold 6654	933,017	928,681 – 933,017	0.050	0.013 – 0.110
scaffold 6654 - scaffold 6680	5,630,660 (6680)	2,536,922 – end (6654) start – 8,158,537 (6680)	0.051	0.004 – 0.090
scaffold 6680	9,994,850	8,849,630 – 14,269,781	0.027	0.003 – 0.087
scaffold 6680	22,866,335	14,677,306 – 24,803,077	0.046	0.019 – 0.111
scaffold 6496	1,817,090	513,803 – 5,902,632	0.090*	0.035 – 0.142
scaffold 6496	20,663,173	15,406,089 – 25,005,286	0.081	0.025 – 0.132

Asterisks indicate that the QTL are antagonistic, wherein the Sonora allele is associated with decreased locomotion and vice versa.

The breadth of the putative QTL on chromosome 3 suggested the potential presence of an inversion. In *D. mojavensis*, inversions have been previously identified on chromosomes 2 and 3 (Ruiz *et al.* 1990). Most recently the inverted region encompassing the inversion of chromosome 3 (3f^2^) has been estimated to be occurring between 6.9 Mb and 16.7 Mb of scaffold 6500 (Guillén *et al.* 2015). To investigate the possibility that an inversion here might be affecting our results, we created new crosses between the two original isofemale lines and photographed polytene chromosomes from F1 salivary glands. Interestingly, chromosome 3 showed no evidence for an inversion polymorphism (Figure S1), which is consistent with long read sequencing data indicating that these two lines are homosequential (C. M. Jaworski and L. M. Matzkin, unpublished data). A second F1 cross, between the Santa Catalina Island line used in this study and a Mojave Desert population from Anza Borrego, CA does reveal an inversion polymorphism (Figure S2), as expected (Delprat *et al.* 2014), suggesting that both parental lines in our cross have the chromosome arrangement 3f^2^. A large chromosomal inversion polymorphism between these two populations, corresponding to 2q^5^ (Ruiz *et al.* 1990; Delprat *et al.* 2014), extends from near the centromeric end of chromosome 2 and spans over half the chromosome (C. M. Jaworski and L. M. Matzkin unpubl data). The QTL identified on chromosome 2 here (Figures 3B, 4) lies within the inversion near its breakpoint. Perhaps due to double recombination events, we do not see evidence for total linkage disequilibrium across the inversion, given the relatively large shift in allele frequencies observed in the relevant region of chromosome 2 (Figure 4). Nonetheless, any suppression of recombination here may inhibit our ability to detect additional significant QTL in this area, as the presence of segregating inversions can be particularly problematic for pooled data (Schlötterer *et al.* 2014).

## Discussion

Our most robust result demonstrates evidence for two relatively large-effect QTL controlling *D. mojavensis* locomotor behavior near the centromeric end of chromosome 3. These regions were identified using all three methods and were estimated by SIBSAM to have the largest effect sizes in the dataset, explaining ∼14% and ∼15% of the variation between bulks. Furthermore, raw allele frequency differences for individual SNPs in the larger of these QTL reached > 0.5, an extremely high discrepancy compared to significant SNPs from studies with similar designs (Swarup *et al.* 2013). The specific location of these regions is notable. A recent meta-analysis of *D. melanogaster* phenotypes from the DGRP populations (Mackay *et al.* 2012) has identified a large clustering of behavior linked QTL near the centromeric end of chromosome 2L (York 2018), including the *foraging* gene (de Belle *et al.* 1989), a protein kinase with major effects on larval feeding and locomotion (Osborne *et al.* 1997). The *D. mojavensis* chromosome 3 is largely homologous to the *D. melanogaster* arm 2L, and though many regions are positionally shifted between the two species (notably including the *foraging* gene itself, for which the *D. mojavensis* ortholog does not lie within either of our QTL), the content of ∼7mb near the centromeric end is reasonably conserved between the two species (Gilbert 2007). This raises the question of whether locomotion in *D. mojavensis* may be in part controlled by an evolutionarily conserved behavioral “hotspot”. The presence of such clusters is also found in mice, where numerous behavioral phenotypes map to a single chromosomal segment (Mozhui *et al.* 2008). Furthermore, complex traits such as behaviors are often underpinned by coadapted gene complexes (Schwander *et al.* 2014; Thompson and Jiggins 2014), wherein behavior associated loci are inherited together due to tight physical linkage or inversions. This question cannot be directly addressed without additional mapping results on *D. mojavensis* behavior, as previous QTL studies on this species (Etges *et al.* 2007; 2009; 2010) utilized a linkage map that unfortunately lacked markers in this area of chromosome 3 (Staten *et al.* 2004). However, we hypothesize that further behavioral traits will indeed map to this segment of chromosome 3.

Both of our methods which identified multiple QTL found support for a minority of these as antagonistic (Rieseberg *et al.* 2002), wherein the allele from the Sonoran Desert population was associated with a lower level of locomotor activity and vice versa. This aligns with previous theoretical (Griswold and Whitlock 2003) and empirical (Rieseberg *et al.* 2002; Albert *et al.* 2008) results on the prevalence of antagonistic QTL. The occurrence of antagonistic effects is attributed to a number of factors, but one factor that may be relevant here is genetic drift (Griswold and Whitlock 2003). The Santa Catalina Island population in particular has experienced a loss of genetic diversity due to a bottleneck (Ross and Markow 2006; Machado *et al.* 2007; Reed *et al.* 2007), and therefore may have fixed small to medium-effect alleles affecting behavior in the opposite direction of prevailing phenotypic trends. Another possible cause for the presence of antagonistic alleles is pleiotropy, wherein selection for correlated characters maintains alleles with otherwise deleterious phenotypic effects (Griswold and Whitlock 2003). Behaviors are expected to display broad genetic correlations with other behaviors (Sih *et al.* 2004), and several behavioral traits in addition to larval locomotion are known to differ between the specific populations under investigation here (Newby and Etges 1998; Etges *et al.* 2006; Coleman *et al.* 2018; K.M. Benowitz, unpublished data).

Though the different statistical methodologies we used painted different pictures of the overall genetic architecture of *D. mojavensis* locomotion, the consensus between them point to a polygenic architecture, with potentially up to eleven loci spread across all chromosomes contributing to phenotypic variation. Furthermore, though effect size estimates from SIBSAM simulations are coarse (Pool 2016), they are at least indicative that several independent loci have large effect sizes for behavioral traits in general as well as locomotor traits, which average under 10% (York 2018). This supports predictions that effect sizes for traits mapped between recently diverged populations should be relatively large (Gavrilets *et al.* 2007; Savolainen *et al.* 2013) and raises the possibility that locomotor behavior might play a role in rapid adaptation to local environments in these populations (York 2018). The Santa Catalina Island and Sonoran Desert populations of *D. mojavensis* are distantly geographically separated, and therefore unlikely to have experienced much migration, as evidenced by measures of gene flow between populations (Smith *et al.* 2012). This may help explain the evidence for persistence of smaller effect QTL in this dataset, which are expected to be lost when gene flow is prevalent (Yeaman and Whitlock 2011), biasing estimates for remaining QTL effect sizes upward (Griswold 2006). What remains unclear, both for this character and many other behaviors, is the direction of causality of the relationship between trait evolution and genetic architecture. Might certain behaviors evolve rapidly due to amenable genetic architectures including large-effect variants, or do these architectures simply reflect the consequences of rapid evolution if behavioral traits experience stronger direct or indirect selection pressures? To answer this question, more studies explicitly comparing the genomic basis of inter and intra-population variation in behavior are needed. Such parallel datasets will shed light on whether behavior is predisposed to rapid evolution in novel environments in part due to the particulars of its molecular underpinnings.
